# Supplementary material for: From sequence to function through structure: Deep learning for protein design
Source: Comput Struct Biotechnol J. 2022 Nov 19;21:238–50. doi: 10.1016/j.csbj.2022.11.014 (PMC9755234; doi:10.1016/j.csbj.2022.11.014)
Supplement: Supplementary data 1 — Supplement 4 contains a glossary of terms for further reading [file mmc1.pdf]

Supplementary material

From sequence to function through structure: deep learning for protein design

Noelia Ferruz, Michael Heinzinger, Mehmet Akdel, Alexander Goncearenco, Luca Naef, Christian Dallago

Supplement 1

380,000 sequences were generated using ProtGPT2 using default parameters: top_k: 950, top_p: 1, temperature: 1, and max_length < 150 on an NVIDIA Quadro RTX 8000 with 48GB vRAM. The process took approximately 3.5h. From these sequences, 180,000 were automatically discarded because they had been truncated during the process (presented with no end-of-sentence tokens). We further ordered them by perplexity, and took the best 100,000 scoring ones. Predictions of functional and structural properties ran on the same GPU in approximately 30 minutes. The U50 dataset was generated by sampling 100,000 sequences from the Uniref50 database. The random dataset was created by randomly shuffling the amino acids in U50 sequences. Prediction of properties for these two datasets ran on the same GPU in ~1.5h/set.

Supplementary material

From sequence to function through structure: deep learning for protein design

Noelia Ferruz, Michael Heinzinger, Mehmet Akdel, Alexander Goncearenco, Luca Naef, Christian Dallago

Supplement 2

Protein sequence of generated ProtGPT2 protein:

```
> seq6621
MARSILVTGANRGLGRGFIRQYLEQPNLHVTACIRDSASPAALDLAARHPGRLVVVELDISDEASVAAARSVLSEHQ
ITHLDVVVANAGLHTAFADLFSFTVDSALKHIDVNTIGTLRLIQALRPLVEKSPQPRFASMSSGMGSVADFTATPTF
NTYGYSVSKASLNMLTAKFHKEESWLKSFAVHPGFVLTDMGGDKAKLTVEEGVVGMVRVIEQATKESTGEFLRYDGT
PLPW
```

Check it online at:

https://embed.predictprotein.org/#/MARSILVTGANRGLGRGFIRQYLEQPNLHVTACIRDSASPAALDLAARHPGRLVVVELDISDEASVAAARSVLSEHQITHLDVVVANAGLHTAFADLFSFTVDSALKHIDVNTIGTLRLIQALRPLVEKSPQPRFASMSSGMGSVADFTATPTFNTYGYSVSKASLNMLTAKFHKEESWLKSFAVHPGFVLTDMGGDKAKLTVEEGVVGMVRVIEQATKESTGEFLRYDGTPLPW

Supplement 3

We underline the potential of natural product space by showing and annotating the chemical space between 48 published molecular glues obtained from iPPIDB and known natural products in bacteria and fungi [1] (**Fig. S1**). We also showcase four examples of glue molecules very similar to four existing natural products (**Fig. S1, panels B-E**). Rapamycin, a macrocycle and BGC product extracted from Streptomyces hygroscopicus, discovered serendipitously on Easter Island (Rapa Nui) and currently used to prevent organ rejection, was identified to inhibit the metabolic master regulator mTOR (mammalian target of rapamycin) with exquisite selectivity by gluing it to the peptidyl-prolyl isomerase FKBP12, thus preventing access to the active site [2]. A non-natural product derived example is the case of lenalidomide, used in the treatment of multiple myeloma and the fifth most selling drug in 2021 [3], which has recently been discovered to act by gluing zinc-finger proteins such as the transcription factor CK1α, a class of proteins considered highly difficult to drug, to Cereblon [4], [5]. Cereblon belongs to a class of protein called E3-ligases which mark proteins with ubiquitin leading to their degradation.

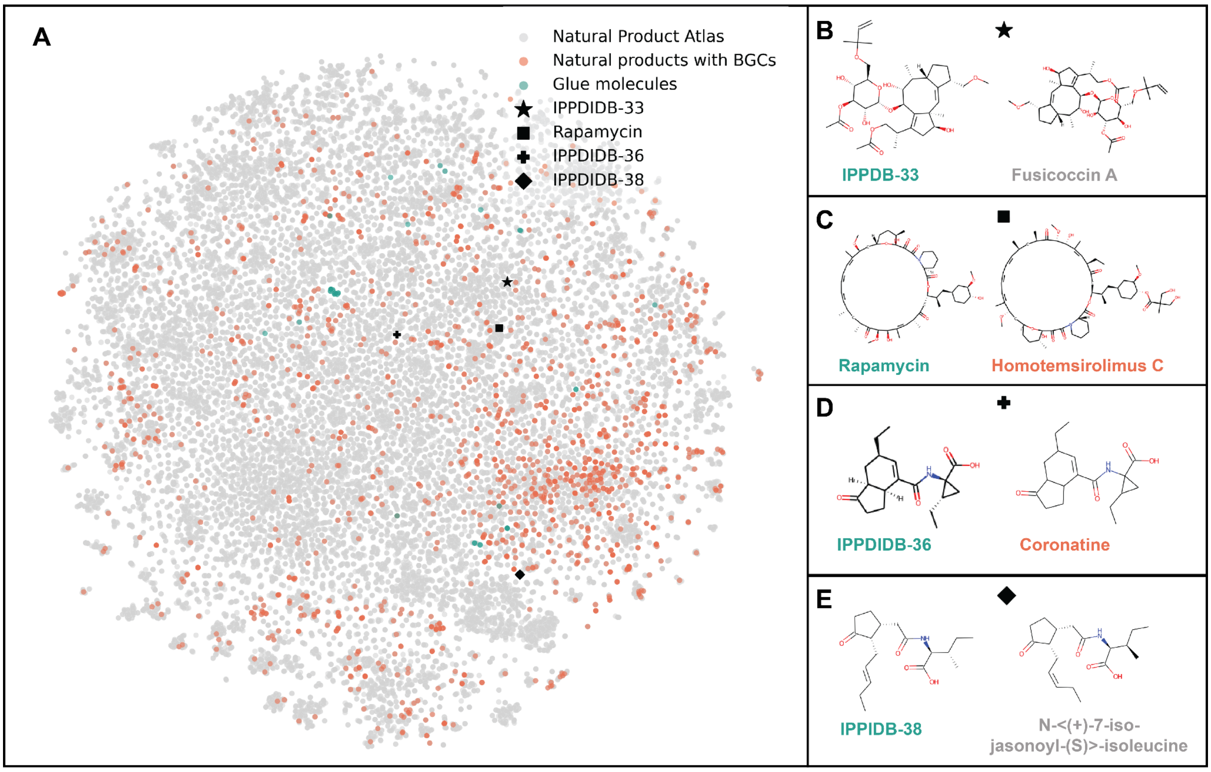

Fig. S1: a projection of t-SNE embeddings of Morgan chemical fingerprints for natural products from the *npatlas* [1] (gray), natural products from biosynthetic gene clusters (orange), and glue-like molecules (green) (panel A). Four examples of glue-like molecules are selected from panel A (marked ★, ■ ,✚ ,♦) cluster along with their closest natural products in panels B through E.

Supplementary material

From sequence to function through structure: deep learning for protein design

Noelia Ferruz, Michael Heinzinger, Mehmet Akdel, Alexander Goncearenco, Luca Naef, Christian Dallago

References

[1] J. A. van Santen *et al.*, "The Natural Products Atlas 2.0: a database of microbially-derived natural products," *Nucleic Acids Res.*, vol. 50, no. D1, pp. D1317–D1323, Jan. 2022, doi: 10.1093/nar/gkab941.

[2] J. Choi, J. Chen, S. L. Schreiber, and J. Clardy, "Structure of the FKBP12-Rapamycin Complex Interacting with Binding Domain of Human FRAP," *Science*, vol. 273, no. 5272, pp. 239–242, Jul. 1996, doi: 10.1126/science.273.5272.239.

[3] B. Buntz, "50 of 2021's best-selling pharmaceuticals," *Drug Discovery and Development*, Mar. 29, 2022. https://www.drugdiscoverytrends.com/50-of-2021s-best-selling-pharmaceuticals/ (accessed Aug. 15, 2022).

[4] J. Krönke *et al.*, "Lenalidomide induces ubiquitination and degradation of CK1α in del(5q) MDS," *Nature*, vol. 523, no. 7559, Art. no. 7559, Jul. 2015, doi: 10.1038/nature14610.

[5] Q. L. Sievers *et al.*, "Defining the human C2H2 zinc finger degrome targeted by thalidomide analogs through CRBN," *Science*, vol. 362, no. 6414, p. eaat0572, 2018, doi: 10.1126/science.aat0572.

Supplement 4

Glossary

Multiple Sequence Alignment (MSA). Starting from a query protein sequence of interest, databases containing protein sequences are searched to find sequences that are similar (i.e. some substring between the query and a database sequence match) to the query protein sequence. After having found potential similar sequences, pairwise alignments, e.g. using the Needleman-Wunsch algorithm, are performed between the query sequence and each of the filtered sequences from the database. Resulting is a matrix of sequences which *align* to the query, from which statistical measurements can be derived, for instance the conservation of one residue across alignable sequences.

Protein Language Models (pLMs) and embeddings. pLMs are language models (LMs) taken from natural language processing but trained on protein sequences instead of natural text. The main advantage of (p)LMs is that they do not require any labelled data during training but instead learn solely from the sequential signal inherent to protein sequences as well as natural text. For natural text, this sequential signal is defined by grammar and syntax while for protein sequences the sequential signal originates from evolutionary pressure that requires functional proteins to have well-defined amino acid patterns. This signal can be picked up by the model, for example, by randomly corrupting tokens, either words or amino acids, from the input and training the network on reconstructing those tokens from non-corrupted input. The knowledge acquired by the (p)LM during this pre-training can be transferred to other tasks by inputting a protein sequence of interest and extracting the hidden states of the p(pLM). Those vectors are also called embeddings and were shown to capture various aspects of proteins.

Evolutionary information. Despite some exceptions, the general trend that proteins with similar sequences fold into similar structures in order to perform similar functions remains true. This idea can be leveraged by grouping proteins into families based on sequence- or structure-similarity. This grouping gives various information, e.g., on the structural plasticity or conservation of a family. Also, co-evolving residues can be traced back with such information if enough related sequences can be found.

Cloze Test. A test where parts of a sentence or document are hidden and a participant is asked to guess the best character, word or phrase to fill the blank. For instance, filling the blank in the sentence "*This morning, I went to _______.*"

Fully-connected neural networks (FNNs), convolutional neural networks (CNNs), graph neural networks, and transformer neural networks. Machine learning architectures that emulate simplified characterization of biological neural networks to achieve a learning objective which may be supervised (e.g. predicting a cat or a dog from an image) or unsupervised (e.g. reconstructing missing parts of an image that are held back from the network during training). FNNs are historically the first popular deep learning network architecture, connecting every input variable to an arbitrary number of intermediate "neurons". These neurons get trained to select which input variables contribute most to an output prediction, for instance learning that some combinations of demographic inputs (e.g. citizenship and year of birth) can help predict a person's current height. CNNs are an evolution to this concept specifically for predictions on images, where local information (e.g. a patch on the top-left of an image of a landscape) may contribute more to a prediction (e.g. the colour of the sky). However, many modalities in the real world are rather 3D objects than 2D images which is why Graph neural networks (GNNs) were introduced to directly learn from graphs or 3D objects that can be represented as graphs such as molecules where atoms can be seen as nodes in graphs. Transformers neural networks are complex architectures which have found success in natural text, biological sequences

and other types of sequential data. These network architectures strive to learn a notion of the ordering of sequences and an underlying, intrinsic meaning of the states within those sequences (e.g. words in sentences or amino acids in protein sequences). On top, they employ the use of mechanisms inspired by human attention, attempting to learn which parts of a sequence contribute the most to understanding its overall meaning and reconstructing its relative constituents.
